# Viable phenotype of ILNEB syndrome without nephrotic impairment in siblings heterozygous for unreported integrin alpha3 mutations

**DOI:** 10.1186/s13023-016-0514-z

**Published:** 2016-10-07

**Authors:** Elisa Adele Colombo, Luigina Spaccini, Ludovica Volpi, Gloria Negri, Davide Cittaro, Dejan Lazarevic, Salvatore Zirpoli, Andrea Farolfi, Cristina Gervasini, Maria Vittoria Cubellis, Lidia Larizza

**Affiliations:** 1 Dipartimento di Scienze della Salute, Università degli Studi di Milano, Via Antonio di Rudinì 8, 20142 Milan, Italy; 2 Genetica Medica, Ospedale Buzzi, Azienda Ospedaliera Istituti Clinici di perfezionamento, Via Castelvetro 32, 20154 Milan, Italy; 3 Dipartimento di Biotecnologie Mediche e di Medicina Traslazionale, Università degli Studi di Milano, Via Viotti 3/5, 20133 Milan, Italy; 4 Center for Translational Genomics and BioInformatics, San Raffaele Scientific Institute, Via Olgettina 60, 20132 Milan, Italy; 5 SC Radiologia e Neuroradiologia Pediatrica, Ospedale Buzzi, Azienda Ospedaliera Istituti Clinici di perfezionamento, Via Castelvetro 32, 20154 Milan, Italy; 6 Dipartmento di Pediatria, Ospedale Buzzi, Azienda Ospedaliera Istituti Clinici di perfezionamento, Via Castelvetro 32, 20154 Milan, Italy; 7 Dipartimento di Biologia, Università degli Studi di Napoli Federico II, Cupa Nuova Cintia 21, 80126 Naples, Italy; 8 Laboratorio di Citogenetica Medica e Genetica Molecolare, Centro di Ricerche e Tecnologie Biomediche IRCCS-Istituto Auxologico Italiano, Via Zucchi 18, 20095 Cusano Milanino, Italy

**Keywords:** Integrin α3, ILNEB variant, Lung disease, Skin alterations, Whole-exome sequencing

## Abstract

**Background:**

Integrin α3 *(ITGA3)* gene mutations are associated with Interstitial Lung disease, Nephrotic syndrome and Epidermolysis bullosa (ILNEB syndrome). To date only six patients are reported: all carried homozygous *ITGA3* mutations and presented a dramatically severe phenotype leading to death before age 2 years, from multi-organ failure due to interstitial lung disease and congenital nephrotic syndrome. The involvement of skin and cutaneous adnexa was variable with sparse hair and nail dysplasia combined or not to skin lesions ranging from skin fragility to epidermolysis bullosa-like blistering.

**Results:**

We report on two siblings of 13 and 9 years born to non-consanguineous healthy parents, who display growth delay, severe pulmonary fibrosis with fatigue, dyspnea on exertion and wheezing, atrophic skin with erythematosus lesions, rare eyelashes/eyebrows and pachyonychia. By exome sequencing, we identified two unreported *ITGA3* missense mutations, c.373G>A (p.(G125R)) in exon 3 and c.821G>A (p.(R274Q)) in exon 6, affecting highly conserved residues in the integrin α3 extracellular N-terminal β-propeller domain. Homology modelling of α3β1 heterodimer fragment, encompassing the mutation sites, showed that G125 plays a pivotal structural role in the β-propeller, while R274 might prevent the interaction between integrin and urokinase complex.

**Conclusion:**

We report a variant of ILNEB syndrome in two siblings differing from the previously reported patients in the lack of nephrotic impairment and survival beyond childhood.

Our siblings are the first reported compound heterozygous for *ITGA3* mutations; this state as well as the hypomorphic nature of their p.(R274Q) mutation likely account for their survival.

**Electronic supplementary material:**

The online version of this article (doi:10.1186/s13023-016-0514-z) contains supplementary material, which is available to authorized users.

## Background

The clinical and genetic heterogeneity of genodermatoses is well exemplified by Epidermolysis Bullosa (EB). Indeed, the EB classification has been recently revised to incorporate the growing list of causative genes and recommends to use a systematic “onion skin” approach which takes into account successive layers of clinical, immunohistochemical and molecular findings [1, 2]. One of the four main EB subtypes, the Junctional EB (JEB), where the cleavage plain of blister formation is within the lamina lucida, comprises forms involving the genes for integrin subunits α6 (*ITGA6,* OMIM*147556), β4 (*ITGB4,* OMIM*147557) and α3 *(ITGA3,* OMIM*605025) [3, 4]. In particular, the *ITGA3* gene has been recently associated to a generalised JEB with respiratory and renal involvement (JEB-RR) or Congenital Interstitial Lung disease, Nephrotic syndrome and Epidermolysis bullosa (ILNEB, OMIM#614748). So far, six unrelated patients with ILNEB syndrome carrying homozygous mutations in *ITGA3* gene are reported [4–7]. All these patients developed in the first months of life severe interstitial lung disease and renal failure leading to death in early infancy, some displayed sparse hair, onychodystrophy and cutaneous alterations, ranging from blistering and skin erosion to epidermolysis bullosa-like phenotype. The slight expression or lack of apparent cutaneous defects might have been not definitive, as these signs are hardly detectable in the first months of life [5–7].

Integrins are transmembrane proteins with a large extracellular portion and a small cytoplasmic domain. Through tightly regulated adhesions with ligands, collectively known as the “integrin adhesome”, integrins mediate cell-to-cell bridges and cell-to-extracellular matrix interactions playing a key role in cell scaffolding and signalling activity [8, 9]. They are obligate heterodimers of α and β chains and 22 different integrins result in mammals from the combination of eighteen α with eight β subunits. In particular, integrin α3β1 is a receptor for laminins, is widely expressed in the epithelia, especially in lung, kidney and skin, and exerts a fundamental role in the structural and functional organization of these multi-compartment organs as proved by disruption of basement-membrane structure and compromised tissue homeostasis of these organs in α3 full- and organ-specific-knockout mouse models [10–14].

Here we report on two 13 and 9 year-old sibs displaying a clinical phenotype resembling that of ILNEB syndrome in the pulmonary and dermatological alterations, but differing in the lack of renal involvement. In the two sibs exome sequencing highlighted a compound heterozygosity for two unreported missense mutations in the *ITGA3* gene, both affecting the integrin α3 extracellular β-propeller domain, although with a different predicted effect. The unique combination of two different *ITGA3* mutations might account for the siblings’ clinical presentation, which may be classified as mild “variant” of the ILNEB syndrome.

## Results

### Clinical reports

**Fig. 1:**
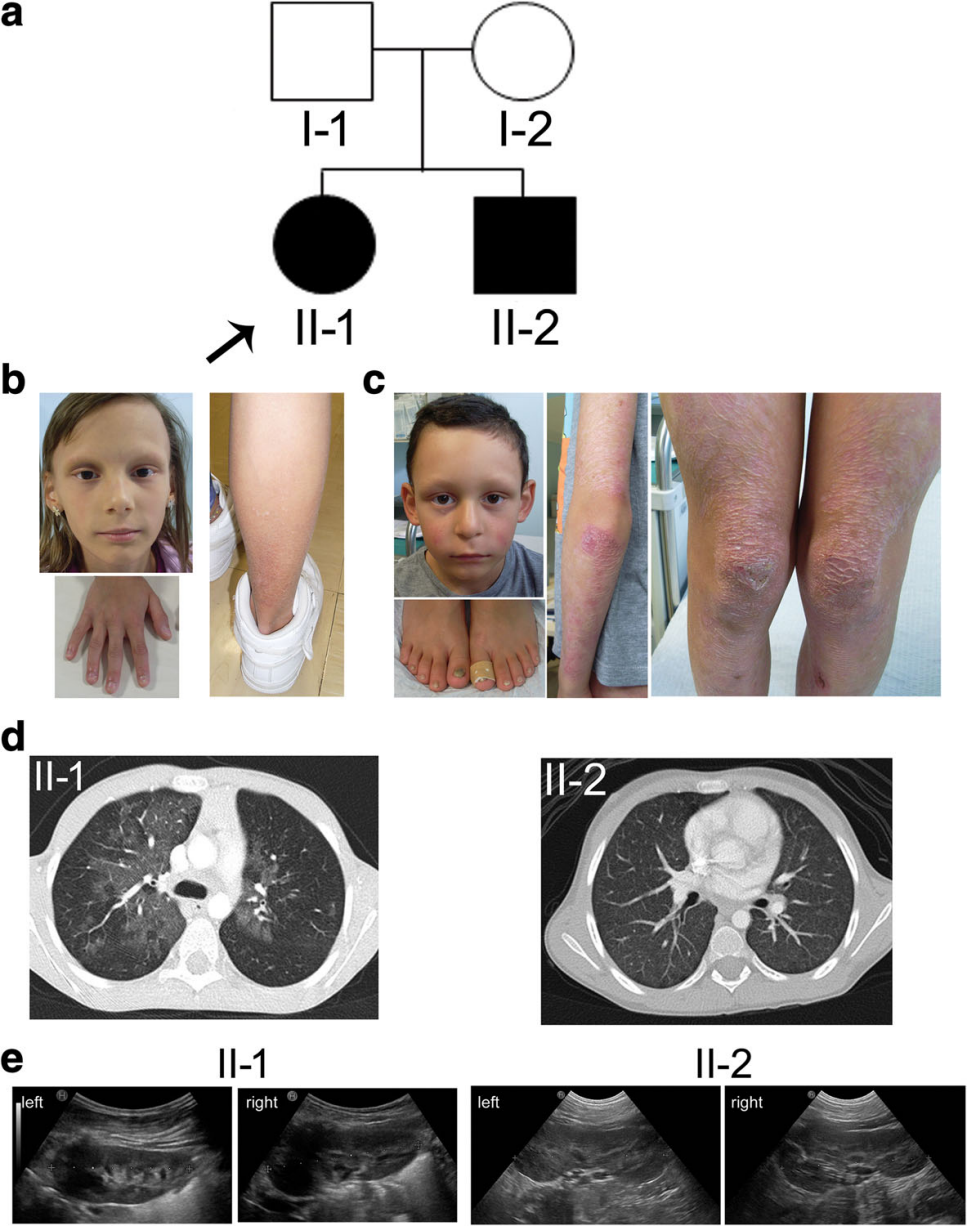
Clinical manifestations in the affected sibs. **a** Pedigree of the patients’ family. The arrow points to the index case. **b** Skin and cutaneous adnexa signs of II-1. To note sparse eyelashes, absent eyebrows, dysplastic nails, atrophic areas and diffuse erythema on the lower part of the leg. **c** Dermatologic changes of II-2. Rare eyelashes and eyebrows, pachyonychia, marked skin atrophy and hypopigmentation with focal areas of erythema at extensor limb surfaces and erosions on the legs are shown. **d** Tomographic scan of the chest of II-1 (7y) and II-2 (5y). Note the diffuse distortion of the pulmonary structure and multiple peripheral areas with reduced density/diffuse ground glass opacity suggesting air-trapping. **e** Renal ultrasound of II-1 (13y) and II-1 (9y) reveals the absence of structural defects in kidneys. A mild asymmetry of the left kidney can be seen for II-1

We report on two affected siblings from a non-consanguineous family presenting with a syndromic phenotype as they shared since the first years of life erythematous skin erosions and respiratory distress with severe air-trapping (Fig. 1). Lung disease is more severe in the elder sister (II-1), while skin involvement is definitely more pronounced in the brother (II-2).

#### Patient II-1

The index case, II-1 (Fig. 1a), is currently 13 years old. Anamnestic records recall an uneventful gestation till the 28^th^ week when foetal growth retardation and oligohydramnios not associated with placental dysfunction were observed. Labour was induced at the 32^th^ week with caesarean section. Her body weight at birth was 2470 g (90^th^ centile) and length 44 cm (75^th^ centile). On day 21^st^, she was hospitalized for sepsis due to *Streptococcus B* infection and was treated with ampicillin and gentamicin. Two days after antibiotics therapy discontinuation, she had a sepsis relapse and angulomandibular adenitis.

She had no major infections in the first years of life, despite severe weight (<3^rd^ centile) and height (3^rd^ centile) deficits. She displayed sparse eyebrows and eyelashes, fine hair, thickened nails and atrophic and erythematous lesions on the legs. Vesicles at popliteal fossae appeared in a few occasions, apparently induced by heating or sweat, but resolved with no visible signs. Both growth delay and dermatologic signs were recorded throughout development (Fig. 1b) and persist up to the present age. Since age 4, she had recurrent respiratory infections and several episodes of pneumonia requiring hospital admissions and she initiated to have breathlessness and severe limitation of day-life activities.

At 6 years of age she had normal oxygen saturation in ambient air at rest, but she had a dramatic effort intolerance, diffuse velcro rales and wheeze. Computed tomography (CT) evaluation showed interstitial pulmonary disease with diffuse mosaic attenuation and minimal calcification in the right para-tracheal localization (Fig. 1d, left).

Lung function test evidenced severe air trapping (residual volume/total lung capacity (RV/TLC) 78.31 (290 %); forced vital capacity (FVC) was initially about 30 %, after prolonged treatment her best was 57 %; forced expiratory volume in the 1st second (FEV1) 30-40 %, O_2_ saturation >97 %). Fiberbronchoscopy was normal and no relevant abnormalities with broncho-alveolar lavage were detected. Ventilation-perfusion scintiscan pointed out an uneven distribution between the lungs (ventilation left 32 % versus right 68 %; perfusion left 16 % versus right 83 %). Since age 8, she reached a stable state which enabled her crossing out from the waiting list for lung transplantation.

At age 11y, bone age study assessed a delay of 1 year; magnetic resonance imaging showed the hypophysis was reduced in size compared to age; low vitamin D and normal IGF1 values were recorded. Growth continued to be stunted with body weight <3°, height at 3°-10° and BMI <<3°. Cardiologic evaluation evidenced slight mitral insufficiency without clinical relevance. Stenosis of the lacrimal ducts with recurrent lacrimation and abnormal teeth eruption were observed. She suffered from gingivitis and oral candidiasis.

Haematological and urine tests, arterial blood pressure and renal ultrasound were repeatedly normal up to the present age (Fig. 1e).

#### Patient II-2

II-2 (Fig. 1a), currently 9 years old, was born at term (2850 g) after an uneventful pregnancy. In the first years of life, he showed sparse and rare eyebrows and eyelashes, pachyonychia at toenails, erythematous lesions on cheeks, chin, neck and limbs and marked skin atrophy with focal areas of hypo/hyper-pigmentation on the neck and limbs. Persistent and multiple erosions involving trauma-exposed skin areas were found at subsequent clinical evaluation (Fig. 1c). Growth parameters were low: at age 3.5y weight was at 25^th^ centile and stature at 15^th^ centile and at 8y weight was at 25^th^ centile, stature at 50^th^ centile and cranial circumference at 10-25^th^ centile.

He had no major infections and normal effort tolerance; chest examination was normal. Pulmonary function test showed air trapping (RV/TLC 42.56 (160 %) and CT scan revealed an interstitial pulmonary disease with air trapping in both lungs (Fig. 1d, right), ventilation-perfusion scintiscan showed minimal inhomogeneity in the lungs (ventilation left 43 % versus right 58 %; perfusion left 44 % versus right 56 %) and O_2_ saturation 98 %.

Cardiologic evaluation at the age of 7y revealed a bicuspid aortic valve and a mild mitral insufficiency. At 9 years of age renal ultrasound is normal (Fig. 1e) and the blood and urine parameters reflecting renal function are all in the normal range.

### Genomic analysis

The sibs were referred to our laboratory following the negative tests for the *CFTR* (OMIM*602421) and *SFTP-C* (OMIM*178620) genes which are implicated in perinatal respiratory distress.

**Fig. 2:**
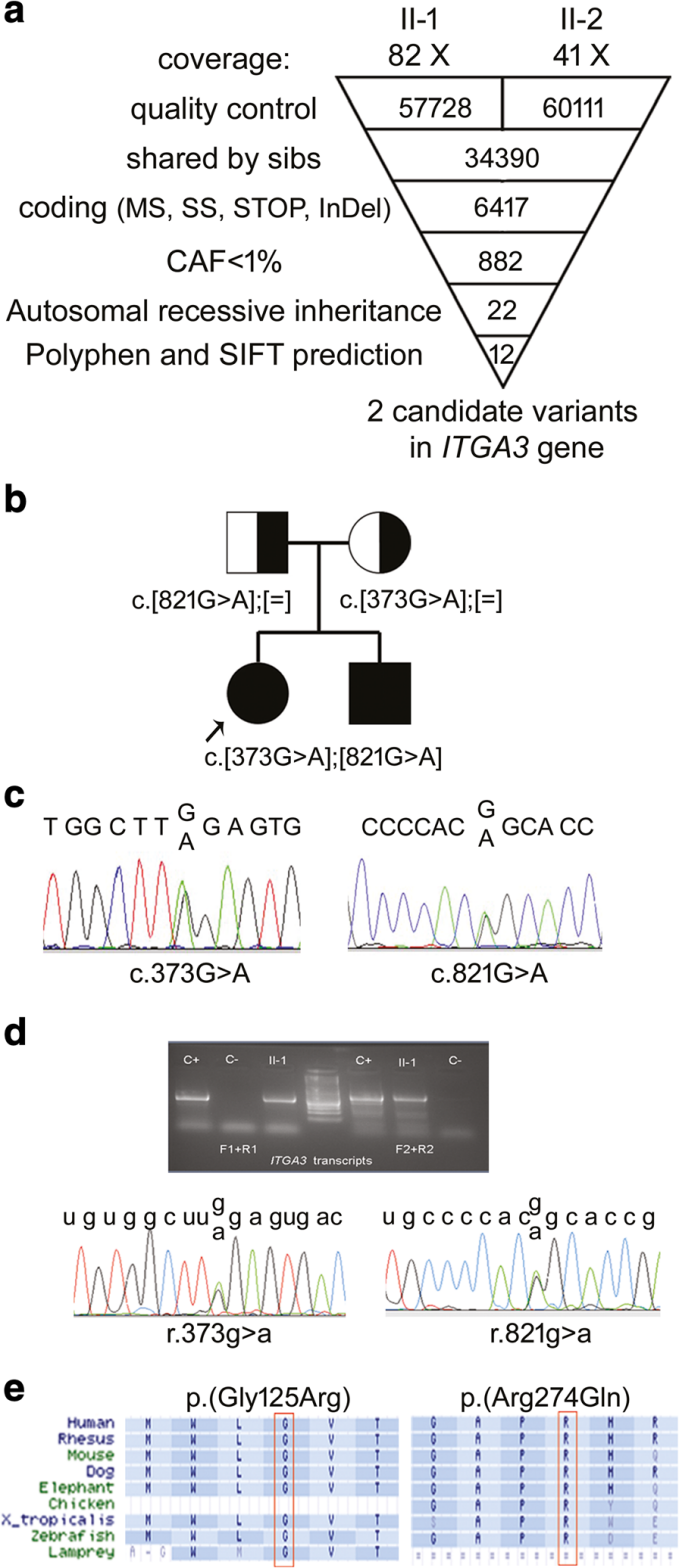
Exome sequencing identifies unreported mutations in *ITGA3* gene. **a** Schematic representation of the exome-data-filtering approach under the assumption of autosomal recessive inheritance of the skin disease with lung involvement in the family. MS: missense; SS: splice site; STOP: nonsense; InDel: insertion and/or deletion. **b** The pedigree of the siblings with the index case: the carrier status of the parents, each bearing a different mutation, and the compound heterozygous affected siblings are indicated. [=]: wild type allele. **c** DNA electropherograms showing the c.373G>A and c.821G>A transitions in the *ITGA3* gene (NM_002204). **d** Agarose gel showing the RT-PCR products amplified by two different primer pairs (F1-R1, F2-R2) in the control cDNA (C+) as well as in the index case (II-1). The sequencing chromatograms of the two fragments spanning the mutation sites are provided below. C-: negative control. **e** Evolutionary comparison across α3 subunit orthologs in nine animal species from human to lamprey shows conservation of the two amino acid residues glycine 125 and arginine 274 (NP_002195) in the siblings family

Due to growth retardation, poikiloderma-like skin lesions on face, neck and limbs, nail dystrophy, hypotrichosis and recurrent infections the *USB1* (OMIM*613276) gene, responsible for Poikiloderma with Neutropenia (PN; OMIM#604173) [15] was tested, but no mutations were detected. Whole Exome Sequencing was then performed on sibs and parents genomic DNA to disclose the causative gene, under the assumption of autosomal recessive inheritance. Following sequence alignment, about 80,000 variants emerged in the whole pedigree and 34,390 were globally found to be shared by the affected sibs (Fig. 2a). Special attention was given to the set of genes involved in autosomal recessive Dyskeratosis Congenita for its clinical overlap with PN [16], but only common reported variants were observed. Subsequent filtering steps, that sorted out variants potentially affecting coding sequences (i.e. nonsynonymous, nonsense or located in the canonical splice-site region) and with a Common Allele Frequency (CAF) <0.01, cut down the number of variants to 882 (Fig. 2a). As no consanguinity has been reported within the family, we selected 22 variants shared by the two sibs, carried by the parents in heterozygous condition and focused on 12 sequence changes, predicted to be damaging by PolyPhen-2 [17] and/or by SIFT [18] score, in 4 candidate genes (Fig. 2a). After gene prioritization, the only candidate gene carrying biallelic alterations and consistent with the clinical phenotype was *ITGA3*. The two different missense changes, c.373G>A (p.(Gly125Arg)) in exon 3, inherited from the mother, and c.821G>A (p.(Arg274Gln)) in exon 6, inherited from the father, were not found in any query of dbSNP-v138 [19], 1000 Genomes Project [20] and 60 in-house controls, except for c.821G>A, which has been recently reported in ExAC [21] browser at 1 out of 120,640 alleles (rs745505565).

Sanger sequencing confirmed that the two sibs were compound heterozygotes and their parents were healthy carriers (Fig. 2b, c). Transcripts analysis evidenced that both mutant alleles were expressed in the patients (Fig. 2d).

The two identified mutations affect amino acid residues, G125 and R274, located in α3 extracellular β-propeller domain, which are highly conserved through evolution (Fig. 2e). The G125 residue is invariant in orthologous alpha chains encoded by the *ITGA3* gene in other species and in all paralogous alpha chains found in humans (α1 to α11, αV, αIIb, αD, αE, αL, αM, αX), while the R274 residue is invariant in orthologous, but not in paralogous alpha chains (data not shown).

### Bioinformatic mutation analyses

Additional file 1 summarises the *in silico* predicted effects of the c.373G>A (p.(Gly125Arg)) and c.821G>A (p.(Arg274Gln)) mutations [see Additional file 1]. In brief the p.(G125R) substitution is deleterious by all the 13 accessed algorithms, while the lower values assigned by most prediction software to p.(R274Q) suggest it might be a hypomorphic mutation.

**Fig. 3:**
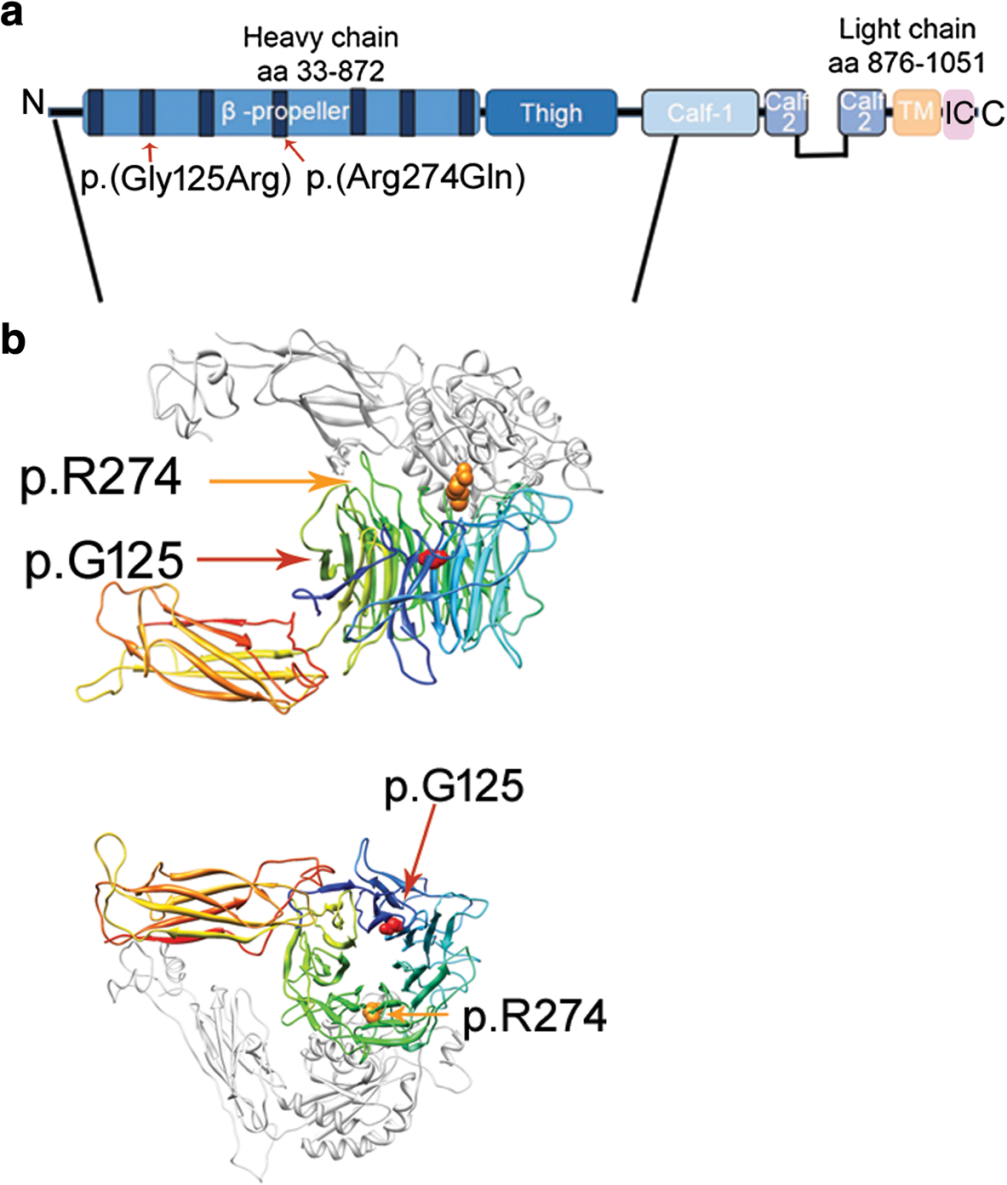
The model of human α3β1 heterodimer. **a** Diagram of the mature integrin α3 subunit. Blue nuances mark the domains of the extracellular portion, the transmembrane domain is orange and the short intracellular domain is pink. The p.(Gly125Arg) and p.(Arg274Gln) fall within the second and the fourth FG-GAP repeats (in black) of the extracellular β-propeller domain. **b** Portion encompassing residues from 34 to 612 of the α3 subunit subjected to 3D modelling is indicated by the diagonal lines. The β-propeller colour ramps from blue (N-terminus) to yellow (C-terminus) and the thigh domain colour ramps from yellow (N-terminus) to red (C-terminus). The plexin/semaphorin/integrin (PSI), hybrid and βA domains of β1 subunit (residues 26-465) are in white. Arrows symbolize beta-strands, curls symbolize helices. Residues G125 (red) and R274 (orange) are rendered in spacefill mode

Furthermore, to make use of predictions concerning the function of the mutant α3 subunits by focussing on most α3 extracellular portion, i.e. the β-propeller, the thigh and part of the calf-1 domain (Fig. 3a), we built a model of the heterodimer α3β1 using a fragment of the human α5β1 ectodomain (Protein Data Bank code 3iv4) [22]. Although not complete, the model shown in Fig. 3b encompasses the sites of the mutations identified in our sibs. G125 (red spacefill in Fig. 3b) occurs in the type II turn that precedes the second blade of the propeller. G125 is buried, has a positive ɸ angle and cannot be substituted by other residues whose lateral chain would cause steric hindrance. This feature together with the highest conservation of this residue in both orthologous and paralogous α chains attests it has a special structural role in the β-propeller domain. R274 (orange spacefill in Fig. 3b) occurs at the tip of the second strand of the fourth blade and interacts with residues of the β1 subunit (white in Fig. 3b). R274 is invariant in orthologous but not in paralogous α chains, suggesting it might be necessary for some specific function of the α3 chain, although it might not play a pivotal structural role in the propeller as G125 does.

## Discussion

We describe two siblings carrying unreported missense mutations in the *ITGA3* gene, which is responsible for Interstitial Lung disease, congenital Nephrotic syndrome and Epidermolysis Bullosa (ILNEB). At difference of the six ILNEB patients so far reported, who all died within the age of 19 months from multi-organ failure, our sibs overcome childhood and are now 13 (II-1) and 9 (II-2) years old, and manifest a mild clinical phenotype due to the lack of overt kidney alterations.

**Fig. 4:**
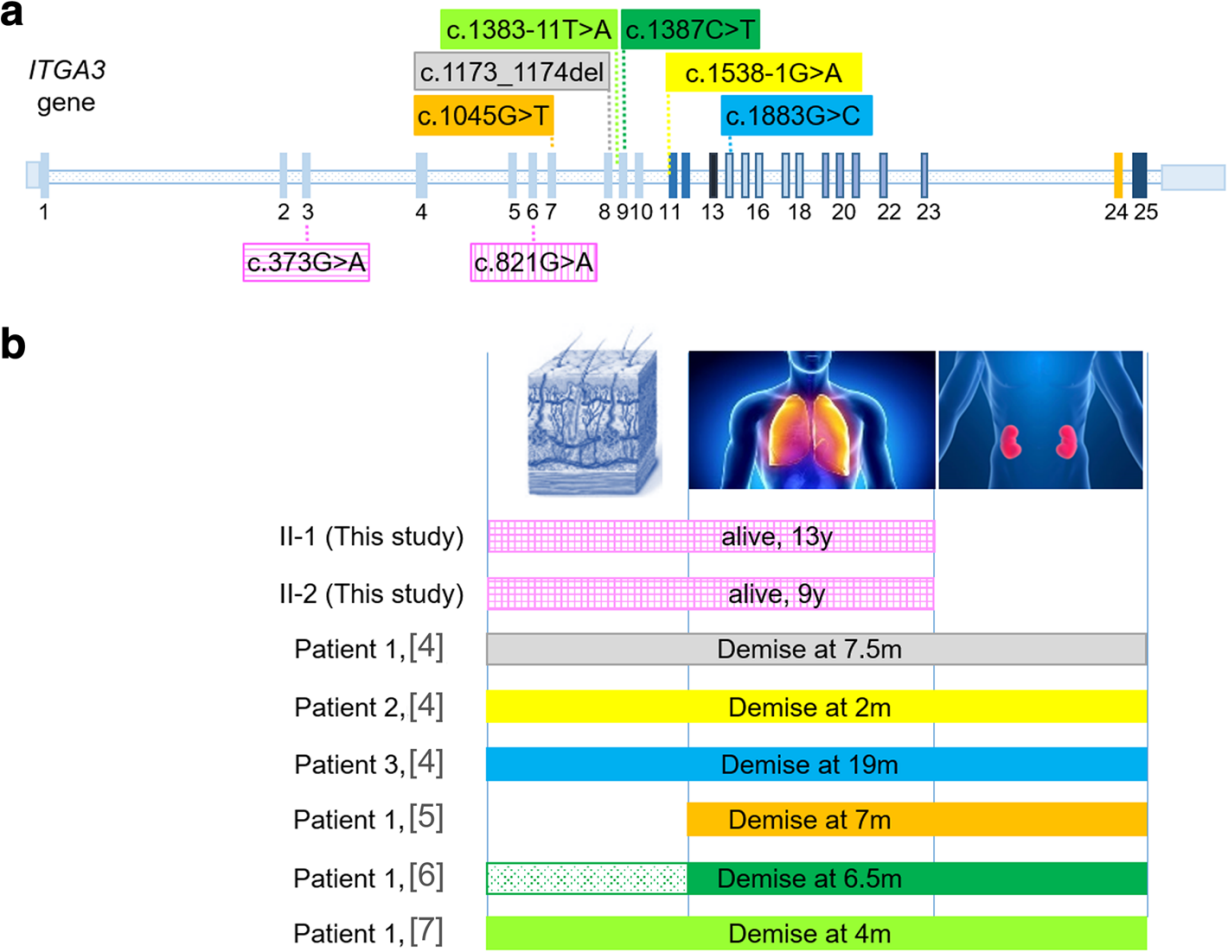
Map of *ITGA3* mutations and related clinical features. **a** Schematic of *ITGA3* gene and localization of the identified mutations. Above the diagram the six reported mutations, all in homozygous state (each framed by a different colour rectangle); below the two different mutations (within rectangles with purples lines) identified in the herein described sibs. **b** The body compartments (skin, lung and kidney) affected in the patients with *ITGA3* mutations. Bars, in the same colour used to highlight the mutations, extend across the three main deranged systems in each patient. The dotted dark green bar of patient 1 [6] indicates the lack of clinical skin disease with the presence of hair and nail signs. Worth noting, no renal involvement is observed in the sibs herein studied, who are the only *ITGA3*-mutated patients surviving beyond the first/second year of life

Figure 4 provides an overview of the mutations identified in all ILNEB patients and their clinical features referred to skin, lung and kidneys involvement.

All patients share a severe early onset interstitial pulmonary disease, documented in our siblings by a distorted pulmonary architecture at CT chest scan (Fig. 1d). Variants in modifier genes and the personal history of the younger brother, starting from birth at term and less impaired growth parameters at difference of his sister, may account for his milder lung disease and his comparatively higher tolerance to physical efforts.

As regards skin involvement, a wide clinical expressivity is observed among the described patients, ranging from the lack of macroscopic skin abnormalities [5] to features such as pachyonychia and fine, sparse hair, eyelashes and eyebrows [6] coupled to mild blistering disorder [4, 7] and erosions [4]. Skin and cutaneous adnexa involvement is present in both our sibs but is more pronounced and diffuse in the brother who evidenced hypo/hyper-pigmentation and erythema with atrophic scarring at multiple sites (face, neck and limbs), and persistent erosion/ulcerations over trauma-exposed areas such as elbows and knees (Fig. 1b).

All six patients with premature demise shared congenital nephrotic syndrome [4, 5, 7] or attenuated renal involvement [6]. Interestingly, concomitant kidney anomalies, ranging from hypoplasia to hypodysplasia and to crossed fused renal ectopia have been found in some cases [4–6, 23] suggesting a central role of *ITGA3* in renal development. Surprisingly our sibs did not manifest signs of renal involvement and only upon the achievement of the genetic diagnosis targeted analyses were carried out and confirmed the normal renal function and the absence of kidney dysplasia/hypoplasia or renal congenital malformations (Fig. 1e). Even if we cannot exclude microscopic kidney alterations, the preserved renal functionality appears a significant contributor to our sibling’ viable phenotype.

A key to justify the clinical variability displayed by the ILNEB patients both across and within the main affected compartments could be represented by the causative *ITGA3* mutations.

The presence of two different alterations with a potentially different functional effect instead of the homozygous mutations detected in all six published cases may contribute to the milder phenotype of our sibs (Fig. 4a). Indeed the paternally inherited R274G is predicted by multiple algorithms and by α3β1 modelling to be a hypomorphic mutation, as the replaced amino acid residue does not play a key role in the correct folding of the α3 chain, even if it might be involved in some α3-specific function. A possible function for R274 is offered by the results of Chapman and co-workers, who found that α3β1 binds urokinase (uPA), a serine protease implicated in extracellular matrix degradation, and its receptor (uPAR) with high affinity [24]. They reported that a peptide derived from α3 encompassing R274, (273-PRHRHMGAVFLLSQEAG-289), can compete specifically and prevent the interaction between the integrin and the uPA/uPAR complex. The specificity of the binding is attested by the fact that homologous peptides either derived from α5 or from αv cannot compete at same way. The uPAR/α3β1 interaction may trigger a pathway of cellular adhesion to vitronectin, especially in cells with little or no αvβ3 [24, 25].

By surveying the functional data obtained on the investigated ILNEB patients, we note that there is a correlation between the age of the demise and the presence of the mature α3β1 heterodimer over the cell membrane. Out of the six characterised patients, five died before 8 months and did not express the α3 subunit. Conversely patient 3 [4], carrying the most distal homozygous mutation, a missense change affecting the calf-1 domain, which has been shown to lead to a residual expression of integrin α3β1 on cell membrane [26], passed away at 19 months.

Although in the case of two different mutations it may be hard to discriminate the different effects of each mutation at the cellular level, we could not perform expression studies due to the unavailability of lung and skin biopsies from our siblings. However, we speculate that at least one mutated protein, likely the one carrying the p.(Arg274Gln) alteration, may be expressed and localized on the cell membrane where it can at least partially work. A tissue-specific penetrance of the “leaky” mutation could explain the lack of renal involvement along with severe lung disease and overt skin anomalies.

In accordance with this hypothesis, a residual protein activity in our sibs might justify their relatively mild overall phenotype and survival till adolescence.

## Conclusion

Our findings on *ITGA3* compound heterozygous patients with an ILNEB-like clinical presentation may account for underestimate of integrin α3 mutated cases and should be kept into account in the processing of exome data from phenotypically candidate patients born to families without consanguinity. Furthermore, the two described siblings may foster better comprehension of the genotype-phenotype correlations in patients with ILNEB and overlapping clinical presentations.

## Methods

The siblings’ parents provided written informed consent to the genetic/genomic test. The study protocol was approved by the Research Ethics Board of ICP, Milan, Italy.

The genomic DNA of the four family members, extracted from peripheral-blood lymphocytes according to standard protocols, was processed for exome sequencing.

Briefly, 2 μg of gDNA was fragmented by Covaris E220, followed by end repair, A-tailing, adapter/barcode ligation and PCR. DNA libraries for each subject have been labelled with a different barcode, pooled and captured together on TruSeq Exome Enrichment preparation kit (Illumina, San Diego, CA) for exome library preparation following manufacturer instruction. Each pool (final concentration of 8 pmol) was sequenced on HiSeq 2500 (Illumina), running SBS 2×101 pair end SBS protocol.

For bioinformatics analysis, read tags were aligned on human reference genome (hg19) using bwa 0.6.1 [27]. After removing duplicate and off-target reads, GATK [28] was used to perform a joint call of SNP and Indels using also a set of 60 exomes of unrelated healthy individuals. Variants were filtered after Variant Quality Score Recalibration and annotated to dbSNP using SnpSift. Functional impact of each variant was predicted using snpEff [29].

Validation of possibly damaging variants detected by exome sequencing in *ITGA3* was performed by Sanger sequencing from PCR amplicons targeted to encompass *ITGA3* variants. PCR was carried out under standard conditions using the following primers (Fex3: 5’-AAGAGGGTGCCCTAGAGGAG-3’, Rex3: 5’-TTGGGAGAGCACAGGATAC-3’; Fex6: 5’-GCTGGCCATCTGGAGTCTAC-3’; Rex6: 5’-CTGCAAACCTCTGCAAACAAC-3’), then the amplicons were sequenced bidirectionally on an ABI3130 DNA Analyzer with BigDye chemistry v1.1 (Applied Biosystems, Foster City, CA). Electropherograms were analysed with ChromasPro software 1.42 (Technelysium Pty Ltd, Tewantin QLD, Australia) using the wild type sequence of *ITGA3* gene (NG_029107.1) as reference. Description of sequence variants has been performed according to HGVS recommendations [30] and *ITGA3* mutations are deposited in the LOVD database [31].

RT-PCR was employed for determining the effect of the detected mutations on transcripts. RNA was isolated using TRI reagent (Sigma, Saint Louis, MI) from EBV-transformed lymphoblastoid cell lines, established from peripheral blood lymphocytes of the elder sib II-1 (SR51711F) and healthy controls and cultured in complete RPMI 1640 medium (EuroClone, Milano, Italy) supplemented with 10 % foetal bovine serum (Lonza, Walkersville, MD) and 1 % Penicillin, Streptomycin and Ampicillin in a 37 °C humidified incubator with 5 % CO_2_. After DNase I treatment (RNase-free, New England Bio-Labs, Ipswich, MA), 250 ng of total RNA were used to cDNA synthesis using the High Capacity cDNA Reverse Transcription Kit (Applied Biosystems) with random hexamers. *ITGA3* cDNA, from exon 1-2 to exon 5-6 (F1: 5’-CGCTACCTGCTCCTGGCTG-3’; R1: 5’-CCTGCATCGTGTACCCAATA-3’) and from exon 3-4 to exon 7-8 (F2: 5’-AGTTCTGGTCTGTGCCCACC-3’; R2: 5’-GCTCCCACAGCAATATCCTGAA-3’) was amplified and sequenced as above described. Nucleotide sequences were compared to the major *ITGA3* transcript reference sequence (NM_002204.2).

To assess the potential impact of *ITGA3* identified substitutions on gene functions, we used different bioinformatic prediction tools: PolyPhen-2 [17], SIFT [18], PMut [32], SNP&GO [33], MutPred [34], SNAP2 [35], PhD-SNP [36], META_SNP [37], Hansa [38], MutationTaster [39], IMutant2 [40], PhastCons [41] and PhyloP [42].

Homology modelling of the heterodimer α3β1 was carried out with MODELLER [43]. The template was the crystal structure of the human α5β1 ectodomain (Protein Data Bank code 3iv4) [22]. We aligned residues 34-612 of integrin α3 to residues 42-642 of integrin α5 with FUGUE [44] and borrowed the structure of integrin β1 residues 26-465 from 3iv4 [22]. FUGUE utilizes environment-specific substitution tables and structure-dependent gap penalties, so that scores for amino acid matching and insertions/deletions are evaluated depending on the local environment of each amino acid residue in a known structure. Dihedral angles and secondary structure were assigned with SEGNO [45]. Figures are drawn with CHIMERA [46].

## Additional file

Additional file 1: Predictions of the effects of *ITGA3* mutation. In this supplementary table we summarize the predictions of the effects of the two *ITGA3* mutations, c.373G >A and c.821G >A, obtained applying different bioinformatic tools. (PDF 117 kb)

## Abbreviations

CT: Computed tomography
EB: Epidermolysis bullosa
ILNEB: Interstitial Lung disease, Nephrotic syndrome and Epidermolysis Bullosa
*ITGA3*: Integrin α3
JEB: Junctional epidermolysis bullosa
