# Supplementary material for: Viable phenotype of ILNEB syndrome without nephrotic impairment in siblings heterozygous for unreported integrin alpha3 mutations
Source: Orphanet J Rare Dis. 2016 Oct 7;11:136. doi: 10.1186/s13023-016-0514-z (PMC5054609; doi:10.1186/s13023-016-0514-z)
Supplement: Additional file 1: — Predictions of the effects of ITGA3 mutation. In this supplementary table we summarize the predictions of the effects of the two ITGA3 mutations, c.373G >A and c.821G >A, obtained applying different bioinformatic tools. (PDF 117 kb) [file 13023_2016_514_MOESM1_ESM.pdf]

Viable phenotype of ILNEB syndrome without nephrotic impairment in siblings heterozygous for unreported integrin alpha3 mutations

Colombo Elisa Adele[1], Spaccini Luigina[2], Volpi Ludovica[3], Negri Gloria[1], Cittaro Davide[4], Lazarevic Dejan[4], Zirpoli Salvatore[5], Farolfi Andrea[6], Gervasini Cristina[1], Cubellis Maria Vittoria[7], Larizza Lidia[8].

[1] Dipartimento di Scienze della Salute, Università degli Studi di Milano, via Antonio di Rudinì 8, 20142 Milano, Italy;

[2] Genetica Medica, Ospedale Buzzi, Azienda Ospedaliera Istituti Clinici di perfezionamento, via Castelvetro 32, 20154 Milano, Italy;

[3] Dipartimento di Biotecnologie Mediche e di Medicina Traslazionale, Università degli Studi di Milano, via Viotti 3/5, 20133 Milano, Italy;

[4] Center for Translational Genomics and BioInformatics, San Raffaele Scientific Institute, via Olgettina 60, 20132 Milano, Italy;

[5] SC Radiologia e Neuroradiologia Pediatrica, Ospedale Buzzi, Azienda Ospedaliera Istituti Clinici di perfezionamento, via Castelvetro 32, 20154 Milano, Italy;

[6] Dipartmento di Pediatria, Ospedale Buzzi, Azienda Ospedaliera Istituti Clinici di perfezionamento, via Castelvetro 32, 20154 Milano, Italy;

[7] Dipartimento di Biologia, Università degli Studi di Napoli Federico II, Cupa Nuova Cintia 21, 80126 Napoli, Italy;

[8] Laboratorio di Citogenetica Medica e Genetica Molecolare, Centro di Ricerche e Tecnologie Biomediche IRCCS-Istituto Auxologico Italiano, via Zucchi 18, 20095 Cusano Milanino, Italy.

Email addresses of all authors:

elisaadele.colombo@unimi.it; luigina.spaccini@icp.mi.it; ludovica.volpi@unimi.it; gloria.negri@unimi.it; cittaro.davide@hsr.it; lazarevic.dejan@hsr.it; salvatore.zirpoli@icp.mi.it; andrea.farolfi@icp.mi.it; cristina.gervasini@unimi.it; cubellis@unina.it; l.larizza@auxologico.it.

Corresponding author: Elisa Adele Colombo, Università degli Studi di Milano, via Antonio di Rudinì 8, 20142 Milano; phone: +39 02 50323200; fax: +39 02 50323026; elisaadele.colombo@unimi.it

Predictions of the effects of *ITGA3* mutation.

	c.373G>A (p.(Gly125Arg))	c.821G>A (p.(Arg274Gln))	Score legend
	Prediction score	***Prediction score***	
**PolyPhen-2** [17]	Probably damaging 1	Probably damaging 1	0.000: most probably benign; 1: most probably damaging
**SIFT [**18**]**	Damaging 0	Tolerated 0.2	score< 0.05: damaging variant
**PMUT [**32**]**	Pathological 1	Neutral 0	score< 0.5: neutral change; score≥0.5: pathological change
**SNP&GO [**33**]**	Disease 7	Disease 5	0:unreliable prediction; 10:reliable prediction
**MutPred [**34**]**	Deleterious 0.924	Deleterious 0.578	0.000: most probably benign; 1: most probably deleterious
**SNAP2 [**35**]**	Effect 71	Effect 66	-100: strong neutral prediction; +100: strong effect prediction
**PhD SNP** [36]	Disease 9	Disease 8	0: unreliable prediction; 10: reliable prediction
**META SNP** [37]	Disease 0.809	Disease 0.641	score>0.5: disease-causing variant
**HANSA [**38**]**	Disease	Disease	
**MutationTaster**[39]	Disease causing	Disease causing	
**IMutant 2 [**42**]**	Stability decrease	Stability decrease	
**Phast Cons [**41**]**	1	0.999	0: most probably not conserved; 1: most probably conserved
**PhyloP** [42]	5.815	5.275	-14: fast-evolving aa; +6: conserved aa
